# Supplementary material for: Antennal Transcriptome Analysis of Olfactory Genes and Characterization of Odorant Binding Proteins in Odontothrips loti (Thysanoptera: Thripidae)
Source: Int J Mol Sci. 2023 Mar 9;24(6):5284. doi: 10.3390/ijms24065284 (PMC10048907; doi:10.3390/ijms24065284)
Supplement: Supplementary file 1 [file ijms-24-05284-s001.zip › ijms-2154638-supplementary.pdf]

Supplementary Materials

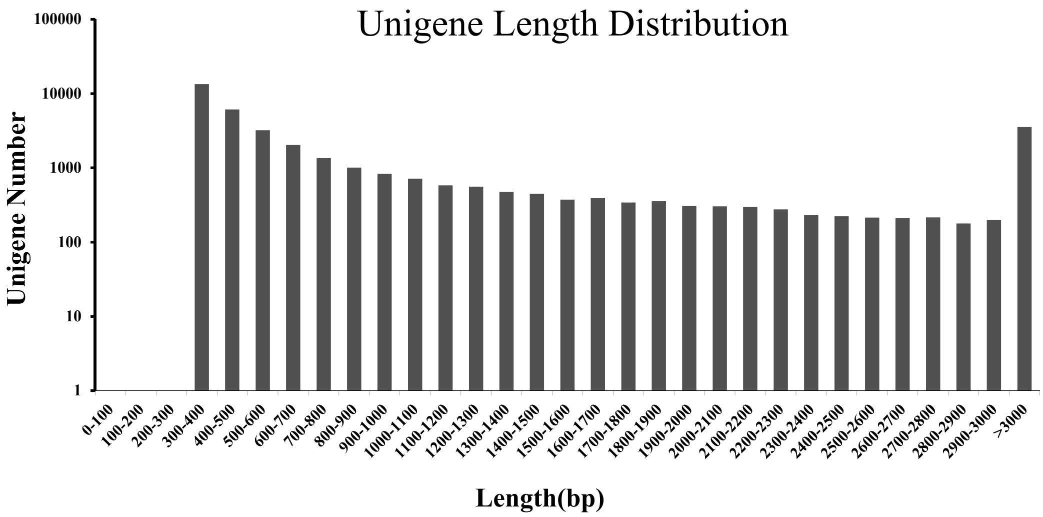

Figure S1. Unigene length distribution graph derived from transcriptome sequencing of the female antennae of *O. loti*.

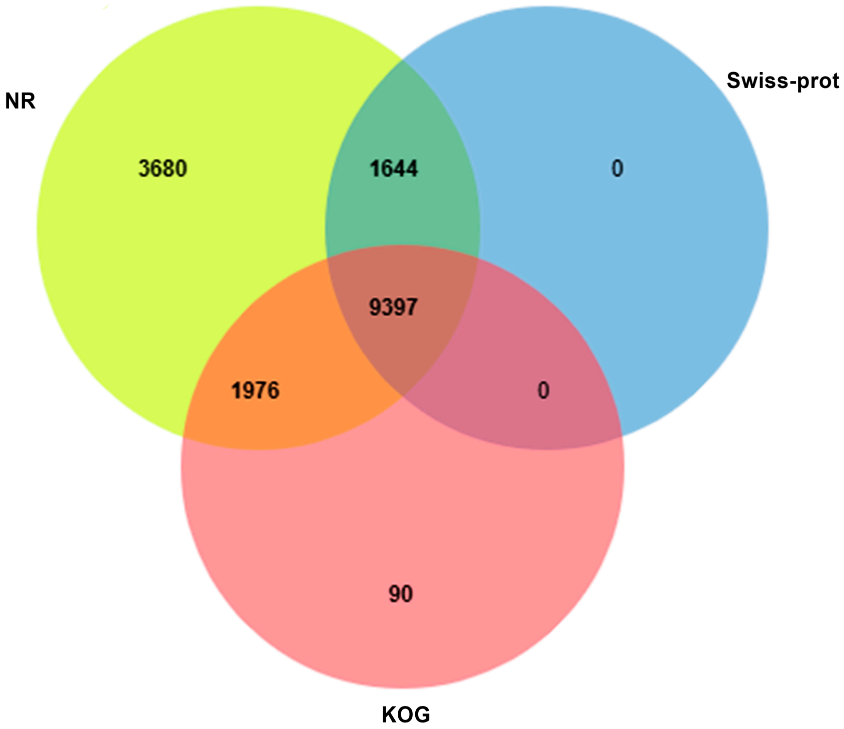

Figure S2. Venn diagram describing the annotated unigenes identified in the three databases (NR, Swiss-prot and KOG) for the antennae of female *O. loti*.

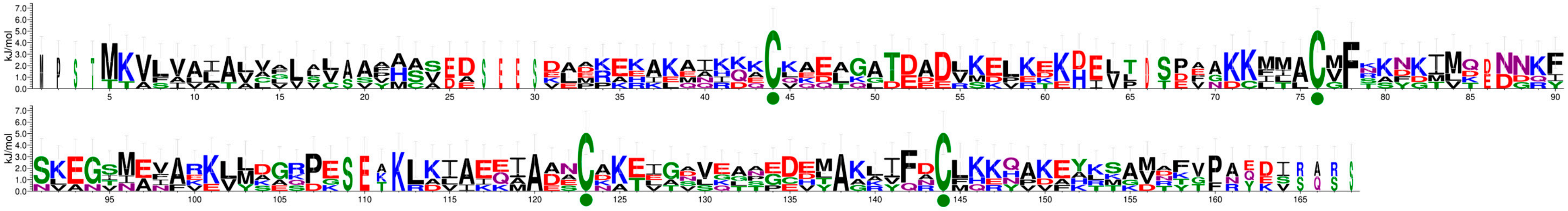

Figure S3. The sequence logo of *O.lot*OBP7 identified from *O. loti* antennae transcriptomes and based on phylogenetic analysis (Figure 2B).

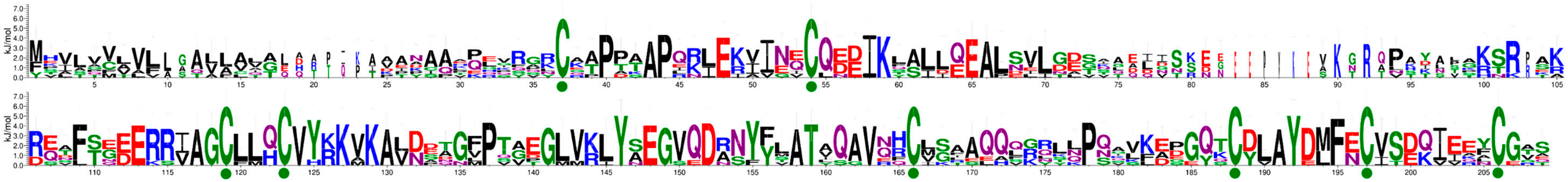

Figure S4. The sequence logo of *O.lot*OBP2 identified from *O. loti* antennae transcriptomes and based on phylogenetic analysis (Figure 2B).

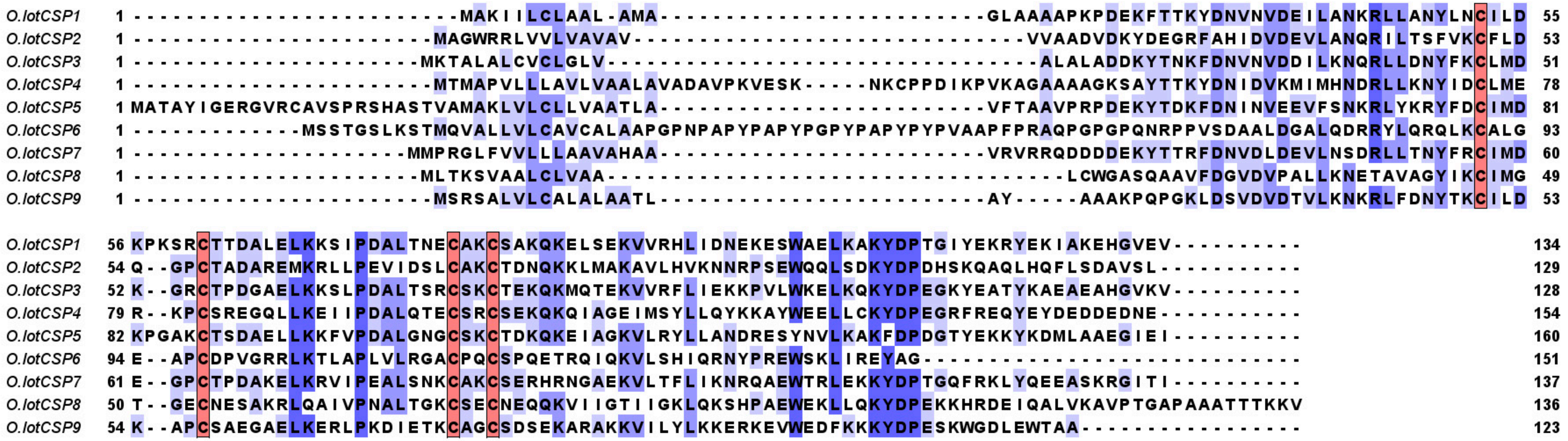

Figure S5. Sequence alignment of *O.lot*CSPs identified from *O. loti* antennae transcriptomes. Red boxes show conserved cysteines, and the color of the purple box shows the strength of homology, the darker the color, the higher the homology.

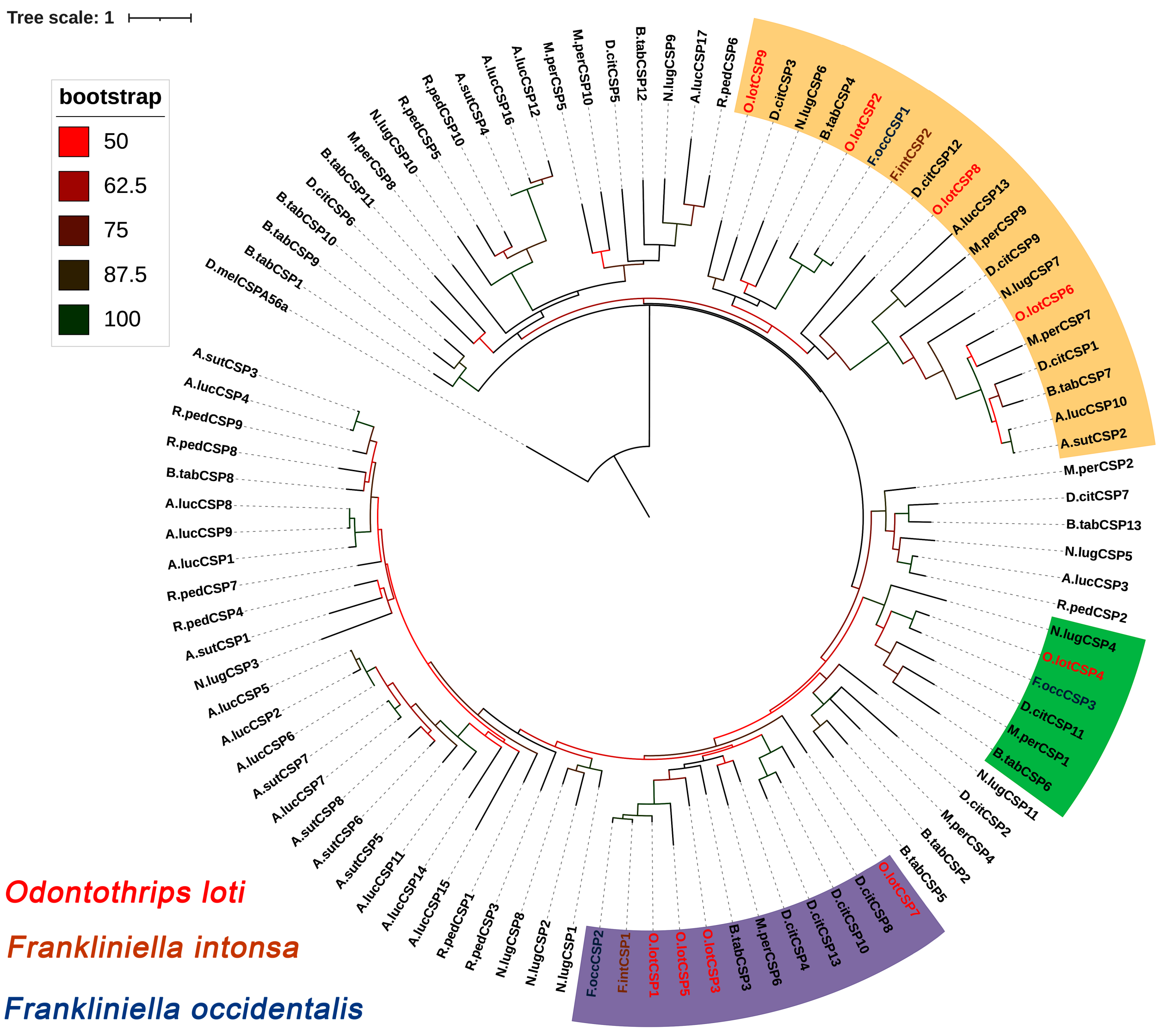

Figure S6. The phylogenetic relationship between the CSPs family and related species of *O. loti*, including *Frankliniella occidentalis*, *Frankliniella intonsa*, *Riptortus pedestris*, *Adelphocoris suturalis*, *Bemisia tabaci*, *Myzus perslcae*, *Apolygus lucorum*, *Nilaparvata lugens*, *Diaphorina citri*, and *Drosophila melanogaster* as an outgroup. The *O.lot*CSPs are highlighted in red.

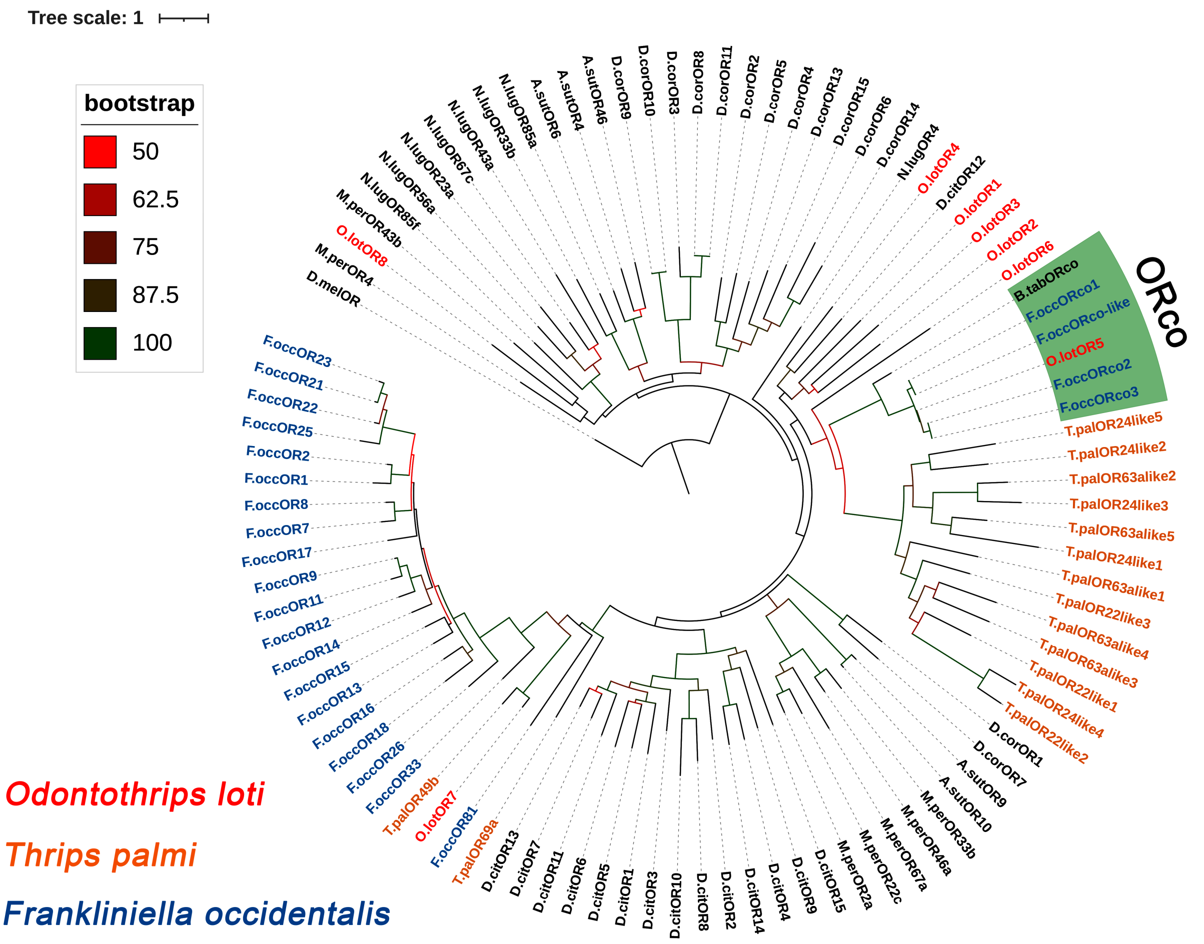

Figure S7. The phylogenetic relationship between the ORs family and related species of *O. loti*, including *Thrips palmi*, *Frankliniella occidentalis*, *Adelphocoris suturalis*, *Bemisia tabaci*, *Myzus perslcae*, *Nilaparvata lugens*, *Diaphorina citri*, *Drosicha corpulenta*, and *Drosophila melanogaster* as an outgroup. The *O.lot*ORs are highlighted in red.

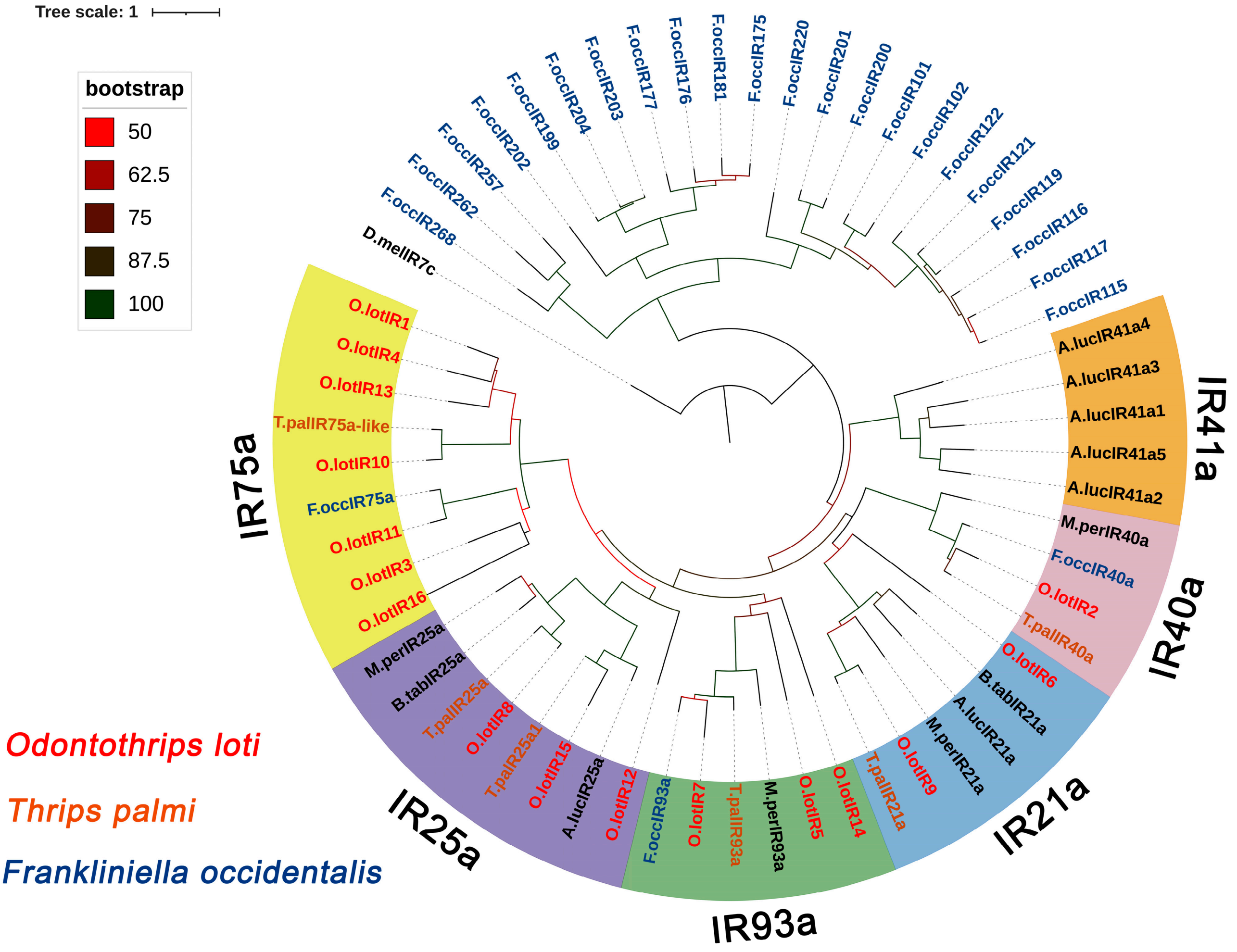

Figure S8. The phylogenetic relationship between the IRs family and related species of *O. loti*, including *Thrips palmi*, *Frankliniella occidentalis*, *Bemisia tabaci*, *Myzus perslcae*, *Nilaparvata lugens*, *Diaphorina citri*, and *Drosophila melanogaster* as an outgroup. The *O.lot*IRs are highlighted in red.

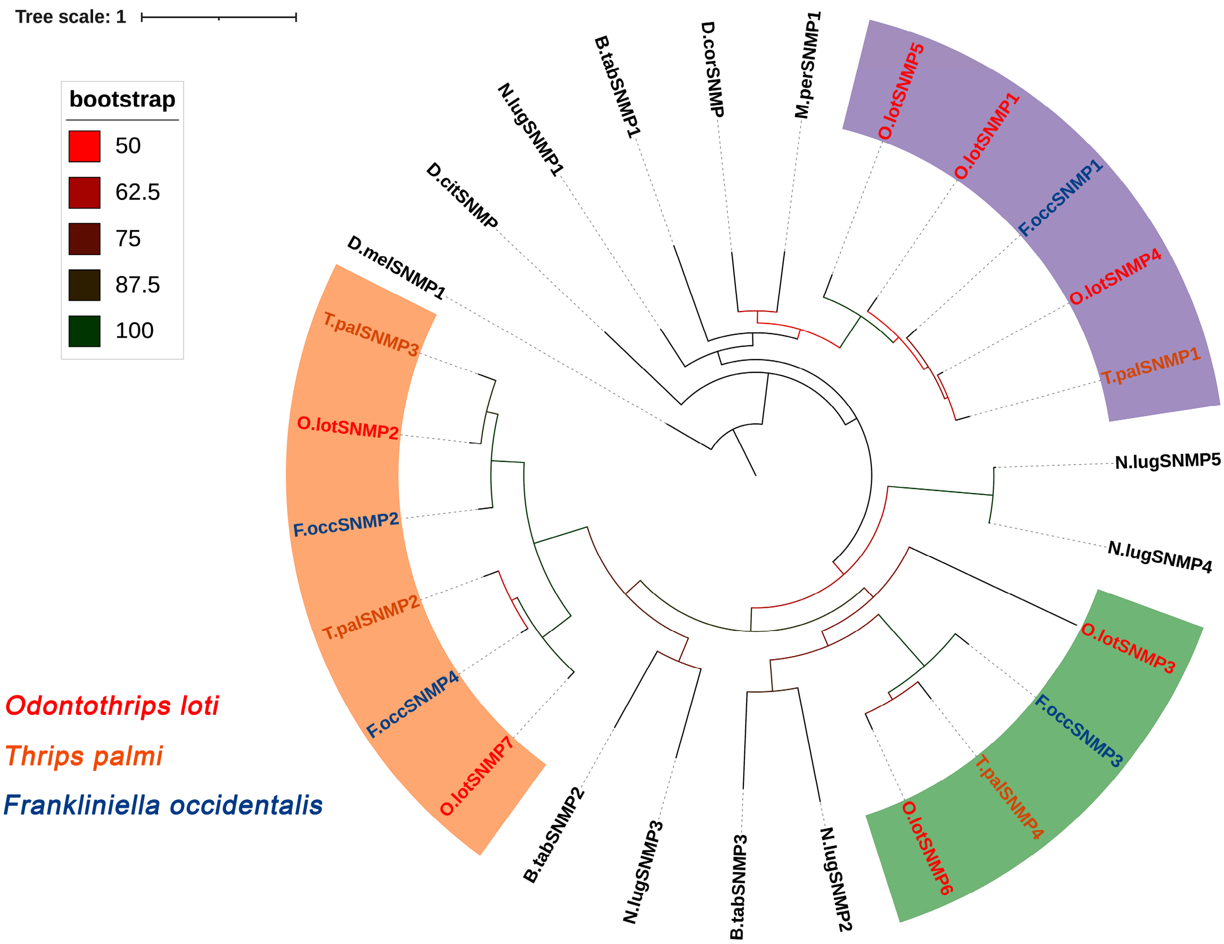

Figure S9. The phylogenetic relationship between the SNMPs family and related species of *O. loti*, including *Thrips palmi*, *Frankliniella occidentalis*, *Bemisia tabaci*, *Myzus perslcae*, *Nilaparvata lugens*, *Diaphorina citri*, *Drosicha corpulenta* and *Drosophila melanogaster* as an outgroup. The *O.lot*SNMPs are highlighted in red.

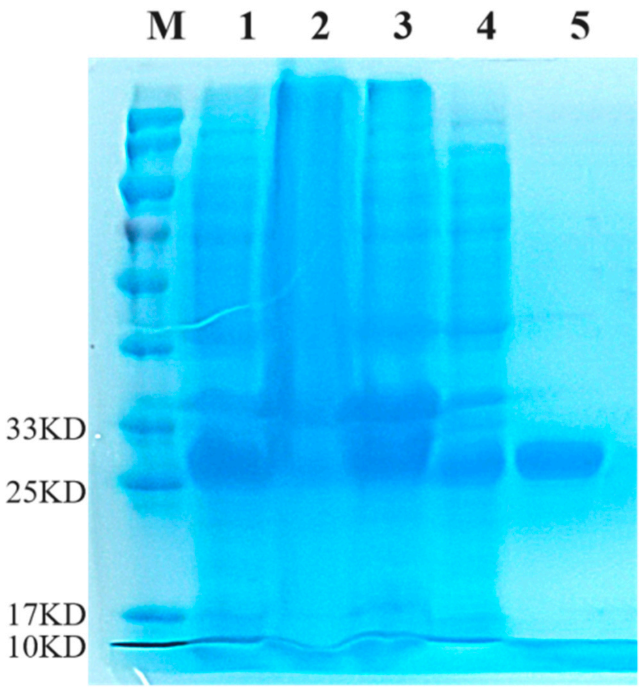

Figure S10. Expression and purification of recombinant *O.lot*OBP6 protein. Protein Marker (M) from top to bottom is 180 KD,130 KD, 95 KD, 72 KD, 55 KD, 43 KD, 33 KD, 25 KD, 17 KD, 10 KD. 1: protein homogenate; 2: protein inclusion body; 3: centrifugal supernatant; 4: protein flow through; 5: purified protein.

A

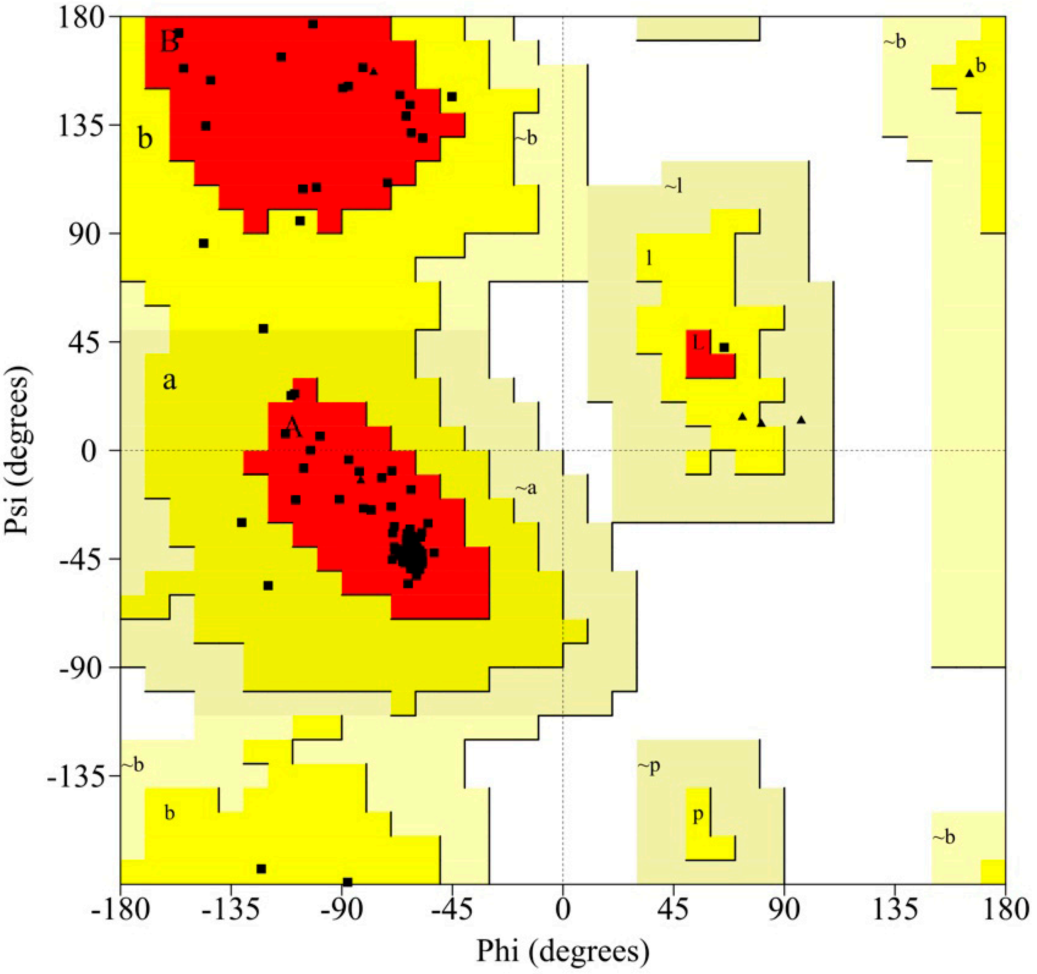

B

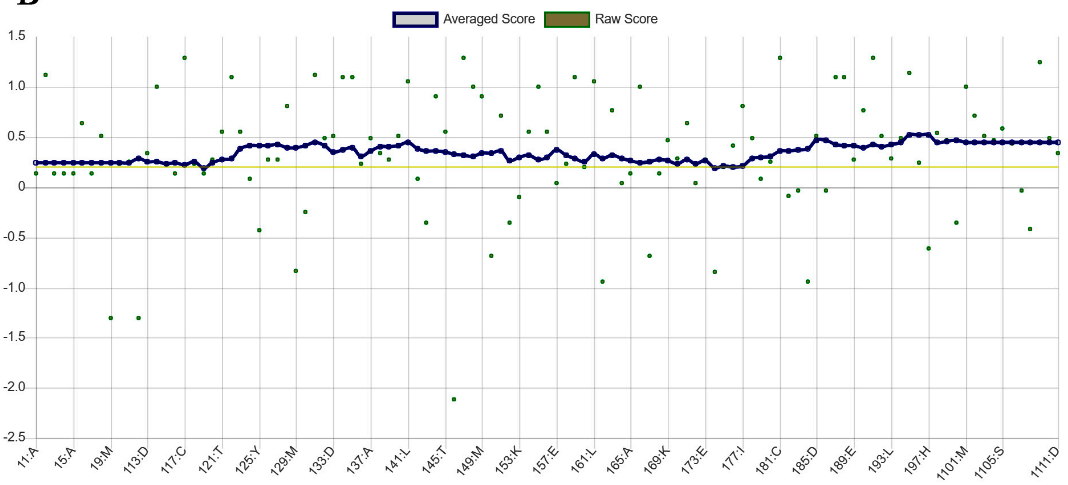

Figure S11. The model quality of *O.lot*OBP6 evaluated by Procheck (**A**) and Verify_3D (**B**). Red areas (A, B, L) represent favoured regions; the yellow areas (a, b, l, p) represent additional allowed regions; the light-yellow areas (~a, ~b, ~l, ~p) represent generously allowed regions, and the white areas represent disallowed regions.

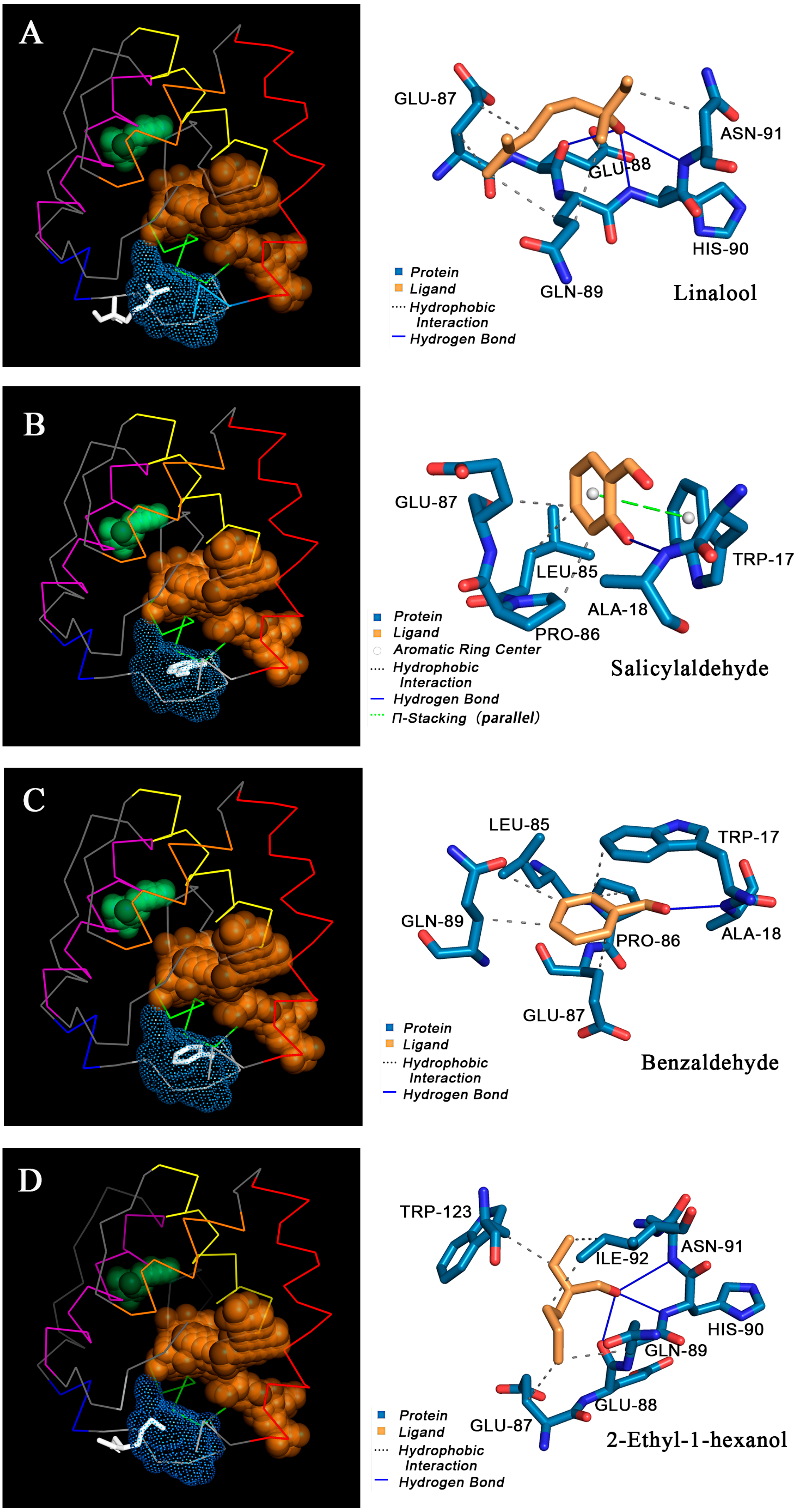
A
GLU-87
ASN-91
GLU-88
HIS-90
GLN-89
Linalool
Protein
Ligand
Hydrophobic Interaction
Hydrogen Bond
B
GLU-87
TRP-17
LEU-85
ALA-18
PRO-86
Protein
Ligand
Aromatic Ring Center
Hydrophobic Interaction
Hydrogen Bond
Π-Stacking（parallel）
Salicylaldehyde
C
LEU-85
TRP-17
GLN-89
PRO-86
ALA-18
GLU-87
Protein
Ligand
Hydrophobic Interaction
Hydrogen Bond
Benzaldehyde
D
TRP-123
ILE-92
ASN-91
HIS-90
GLN-89
GLU-88
GLU-87
Protein
Ligand
Hydrophobic Interaction
Hydrogen Bond
2-Ethyl-1-hexanol

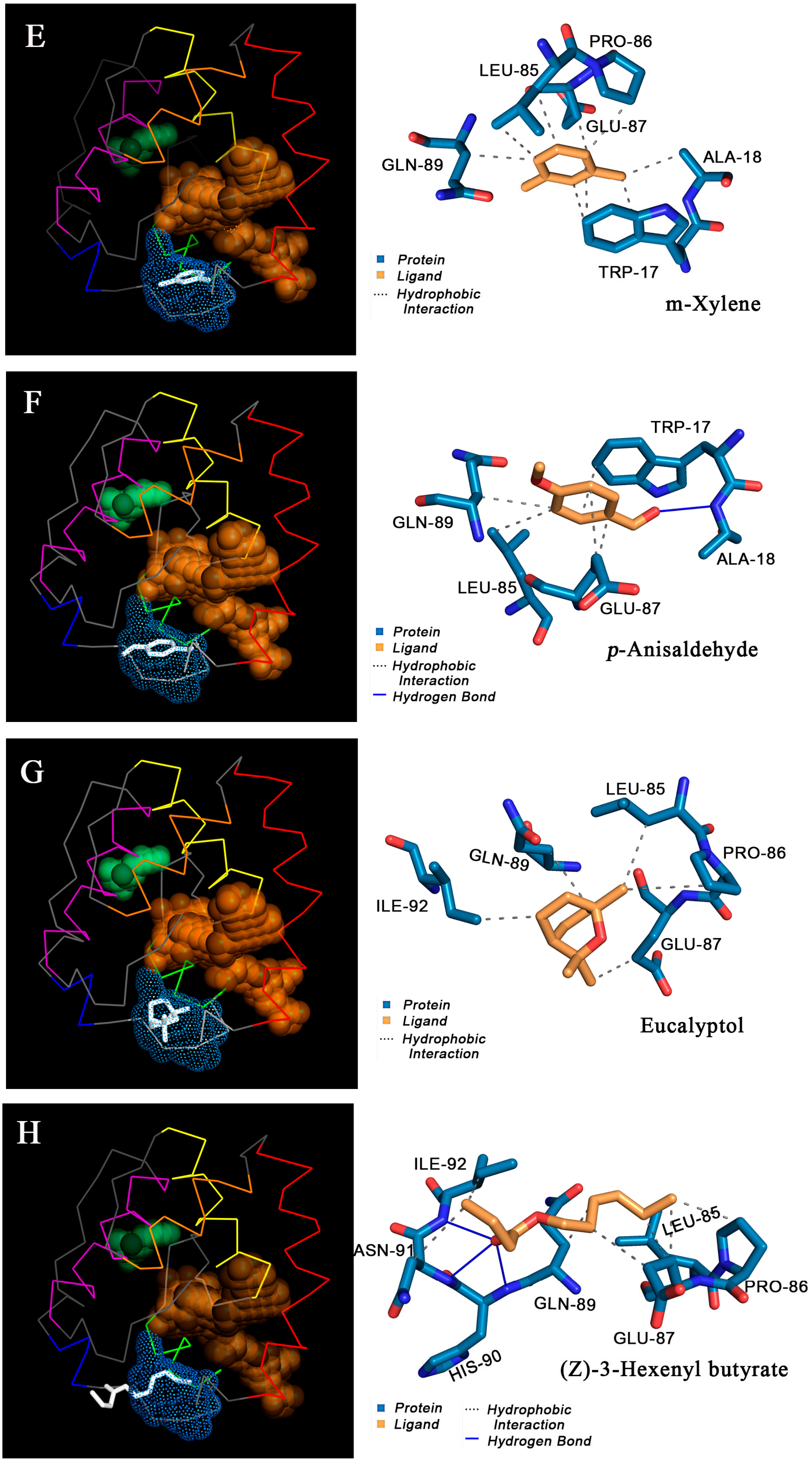

Figure S12. Molecular docking of O.lotOBP6 with 8 ligands, including linalool (**A**), salicylaldehyde (**B**), benzaldehyde (**C**), 2-ethyl-1-hexanol (**D**), m-Xylene (**E**), *p*-Anisaldehyde (**F**), eucalyptol (**G**) and (Z)-3-hexenyl butyrate (**H**). Ligand molecules are shown in white (left)/yellow (right), and amino acid residues are shown in blue (right). The six α helices of O.lotOBP6 are represented by lines of different colors, consistent with those in Figure 4A.

Table S1. The assembled RNA-seq resulting from transcriptome sequencing of female antennae of *O. loti*.

Assembly Method	Length/bp	Unigene Number	Mean Length/bp	>1kb Number	N50/bp
Trinity	42,615,686	38,276	1,113	10,404	2,294

Table S2. Summary of odorant receptors (ORs) and sensory neuron membrane proteins (SNMPs) sequences identified in female antennae of *O. loti*.

Gene Name	Gene ID	GenBank acc.number	ORF (bp)	Complete ORF	TMD (No.)	Homology search with known proteins			
						Species	Acc.number	E-value	Identity
*O.lot*OR1	c40609.graph_c0	OM732445	1065	Yes	4	*Locusta migratoria*	ALD51480.1	3×10^{-16}	23.91%
*O.lot*OR2	c40657.graph_c0	OM732446	1263	Yes	4	*D. melanogaster*	NP_525046.1	2×10^{-7}	24.46%
*O.lot*OR3	c42854.graph_c0	--	1038	Yes	4	*Schistocerca gregaria*	ASM47150.1	7×10^{-4}	25.75%
*O.lot*OR4	c43475.graph_c0	OM732447	651	Yes	2	*D. melanogaster*	NP_523721.1	3×10^{-4}	25.71%
*O.lot*OR5	c46542.graph_c0	OM732448	1506	Yes	7	*F. occidentalis*	XP_026285623.1	0	93.05%
*O.lot*OR6	c44145.graph_c0	OM732449	897	Yes	1	*F. occidentalis*	AKF17721.1	2×10^{-100}	57.29%
*O.lot*OR7	c39377.graph_c0	OM732450	1122	Yes	5	*T. palmi*	XP_034253719.1	1×10^{-52}	63.57%
*O.lot*OR8	c41308.graph_c0	OM732451	1212	Yes	4	*Plutella xylostella*	XP_037970628.1	2×10^{-7}	33.33%
*O.lot*SNMP1	c44520.graph_c1	OM732452	1233	No	1	*Tribolium castaneum*	D2A0H5.1	7×10^{-122}	45.50%
*O.lot*SNMP2	c45512.graph_c0	OM732453	1491	No	1	*T. palmi*	XP_034249405.1	0	80.47%
*O.lot*SNMP3	c45558.graph_c0	OM732454	1782	Yes	2	*Ostrinia nubilalis*	E5EZW9.1	3×10^{-34}	28.04%
*O.lot*SNMP4	c45590.graph_c0	OM732455	1506	Yes	2	*Apis mellifera*	P86905.1	2×10^{-157}	45.28%
*O.lot*SNMP5	c47261.graph_c0	OM732456	1344	Yes	1	*F. occidentalis*	XP_026294183.1	0	68.55%
*O.lot*SNMP6	c15982.graph_c0	OM732457	771	Yes	1	*F. occidentalis*	XP_026287406.1	3×10^{-97}	72.38%
*O.lot*SNMP7	c39262.graph_c0	OM732458	1191	No	1	*F. occidentalis*	XP_026289421.1	0	78.88%

TMD: transmembrane domain.

Table S3. Summary of ionotropic receptors (IRs) sequences identified in female antennae of *O. loti*.

Gene Name	Gene ID	GenBank acc.number	ORF (bp)	Complete ORF	Signal Peptide	TMD (No.)	Homology search with known proteins			
							Species	Acc.number	E-value	Identity
*O.lot*IR1	c8768.graph_c0	OM732459	759	Yes	0	2	*F. occidentalis*	KAE8737063.1	3×10^{-73}	69.68%
*O.lot*IR2	c25526.graph_c0	OM732460	756	Yes	1-22	0	*T. palmi*	XP_034250854.1	7×10^{-117}	89.62%
*O.lot*IR3	c34694.graph_c0	--	1185	Yes	0	3	*L. migratoria*	ALD51365.1	3×10^{-60}	34.91%
*O.lot*IR4	c34826.graph_c0	OM732461	966	Yes	1-27	2	*Drosophila sechellia*	A0A1J0M738.1	2×10^{-4}	30.77%
*O.lot*IR5	c38458.graph_c0	OM732462	1284	Yes	0	3	*O. asiaticus*	QAB43875.1	9×10^{-97}	47.24%
*O.lot*IR6	c40533.graph_c0	--	1377	No	0	3	*Blattella germanica*	PSN33061.1	1×10^{-100}	46.18%
*O.lot*IR7	c42649.graph_c0	OM732463	1095	Yes	0	2	*Anoplophora chinensis*	AUF73071.1	2×10^{-79}	48.32%
*O.lot*IR8	c42714.graph_c0	OM732464	1725	Yes	0	3	*Dendrolimus houi*	AII01111.1	0	55.76%
*O.lot*IR9	c45726.graph_c0	OM732465	2385	No	1-25	3	*Cephus cinctus*	ARN17850.1	6×10^{-140}	41.75%
*O.lot*IR10	c45853.graph_c0	OM732466	2352	Yes	1-18	3	*Heliconius melpomene*	AMM70652.1	1×10^{-77}	30.26%
*O.lot*IR11	c46973.graph_c0	OM732467	1977	Yes	1-37	4	*F. occidentalis*	KAE8736908.1	0	69.20%
*O.lot*IR12	c47462.graph_c0	OM732468	3081	Yes	1-29	4	*Aphidius gifuensis*	AZQ24969.1	0	74.79%
*O.lot*IR13	c34826.graph_c1	OM732469	639	No	0	0	*F. occidentalis*	KAE8739734.1	1×10^{-74}	77.70%
*O.lot*IR14	c44903.graph_c0	OM732470	1731	Yes	1-38	0	*A. gifuensis*	AZQ24985.1	0	64.95%
*O.lot*IR15	c41239.graph_c1	OM732471	1590	No	0	0	*F. occidentalis*	XP_026275220.1	3×10^{-162}	84.53%
*O.lot*IR16	c46726.graph_c0	OM732472	1743	No	1-24	3	*D. abietella*	QJX59526.1	3×10^{-14}	24.61%

TMD: transmembrane domain.

Table S4. Primer sequence of PCR for *O. loti*.

Gene	Forward primer (5'-3')	Reverse primer (5'-3')	Product Size(bp)
*O.lot*OBP1	GTCGTCGTCCATCACCGTCATT	GCTGATGAAGCAGTTGCGTGAG	154
*O.lot*OBP2	GCCGTCCCTTCACTTCCTT	CATCAACGAGTGCCTGGAG	101
*O.lot*OBP3	AAGACTGGAGCGACTGATGAA	GCCTTGTCCTCGTCAACCT	140
*O.lot*OBP4	GCAGGAAGCAAGCAGTGTAGCA	TGACGCAGGAGCAATACGAGAA	150
*O.lot*OBP5	ATATGCCAGCCGAAGTCAGGAG	GAGACCAGAACACAGCCGATGT	110
*O.lot*OBP6	TGGCAATGTTGTGCTGCTCCT	TGCTCCGCTGTAAGACGATGAC	90
*O.lot*OBP7	TCTGTCGTGACTTCGTGCTTCT	CCGTGGACGATGACGAGATGAA	121
*O.lot*CSP1	GCGGACCACCTTCTCAGACA	CGCCTGCTCGCTAACTACCT	159
*O.lot*CSP2	GCAGTGCATGGTCCTTGGTCAA	GCTAGTGGCGGTTGCTGTAGTG	150
*O.lot*CSP3	AGCCGCTGCTCCAAGTGTA	GCCTTGTAGGTCGCCTCGTA	143
*O.lot*CSP4	TGTCCTCGTCGTCTTCGTCGTA	GCTGGCGAGATCATGTCCTACC	124
*O.lot*CSP5	TGTTGCTGAAGACCTCCTCCAC	CTCGTTCTCTGCCTGCTGGT	121
*O.lot*CSP6	ACCTCCAGAGGCAGCTCAAGTG	TGGATGTGGGACAGCACCTTCT	157
*O.lot*CSP7	GCAGCGGAAGTAGTTGGTGAGC	CAGGACGACGACGACGAGAAGT	99
*O.lot*CSP8	GCCGATGATGGTGCCGATGAT	AGTGCAACGAGTCCGCCAAG	110
*O.lot*CSP9	CGCACTTGGTCTCGATGTCCTT	CGCCTGTTCGACAACTACACCA	106
*O.lot*OR1	CTGCGTCTGATGCTGATGAT	GGAGTTGGAGCAGATGAGGA	135
*O.lot*OR1	TGTTGTTCGGCATGACTTACG	GAGAGGCAGGAGTTGTTGGA	179
*O.lot*OR2	TGTTCCTCAGCAGCACCAT	TTCTTCTTCGCCTCGTCTGT	136
*O.lot*OR3	TTGCTGGAACAACCACTGAAG	ACGAAGACGAACGAGATAGAGG	164
*O.lot*OR4	CGGAGAACGAGAACGCCACCAT	CCAGCATCACCAGCAGCCAGTA	138
*O.lot*OR5	CGAGGCGGCTCATGGAAAGTT	TGAGGAGGCATGTCAAGCAGTG	138
*O.lot*OR6	CACACCAAGTACCACAGCAC	CTTCTTCGTCGTCACCATCC	111
*O.lot*OR7	GTAGCACAAGACGAAGATGGT	GGTGGAGCAAGGACATGGT	176
*O.lot*OR8	CATACAGGTGCGGCAGTGA	GTTCCAGGATGAGGTCGTCTT	188
*O.lot*SNMP1	GGAACAGACCCTCGCTGACAGA	CGGACCACCAGACCATCGTTGA	138
*O.lot*SNMP2	CGAGCGAGAACAAGGAGAACCA	GGAAGTGCGGGAACGAGAAGAG	126
*O.lot*SNMP3	GAGTCGGACAGTAGCATCGT	TCAGAAGGAGGTGGTGTTCAA	101
*O.lot*SNMP4	CGTGCGTTGCGAAGAGGTGT	ATGAAGTGCCTTTGCCCGACTC	115
*O.lot*SNMP5	TCCACGGCGTCCAGGTAGAA	GGCGGAGAACGGCAACAACTA	174
*O.lot*SNMP6	CGTCCTCGTCAACTGCTCGTA	AGGTGGCTGAACATGGAGAAGT	132
*O.lot*IR1	CCATCATCCAGTCGCCAAGCAA	CCTCCCGAGTGACAACGAAACC	198
*O.lot*IR2	TCCGACTCCTCACCATCCTCCT	AGCTGCTCCAGGTTGCTGATG	127
*O.lot*IR3	CTGTACCGCCACAAGATCCT	CGATGAGTCCTCCGAGTAGAAG	114
*O.lot*IR4	TTCTCCACGGTCACGATGAT	AACGAAGCCTACCACGAGAT	110
*O.lot*IR5	CTGCTGGAGGCTGGTATCA	CTGCGAAGAAGGCGATGAC	172
*O.lot*IR6	TTCCTCCTCCTCCTTGTCATC	GCGTCAGAGCAACCTTCAG	191
*O.lot*IR7	AACGGCTTGATGACCTTCTTG	GTGCTGTTCTTCGGCTTCC	101
*O.lot*IR8	CTGCGGCGTGAAGGAAGTCA	GTGATGGTGTGGCTGCTGGA	126
*O.lot*IR9	AGAAGTAGGCGTGCGTCAT	GGTCCACCTGGTTCCTGAAT	114
*O.lot*IR10	GCGTATTGCTCGATGAAGTAGA	GCCGTGCTCCTGCTGTATA	158
*O.lot*IR11	GCGATCTTCTCGTCGTAGAG	ACCTTCCTGCTCACCTTCC	158
*O.lot*IR12	TACACGAGCGGCTTCTACC	CGACGAGTGGATGAAGATGAC	183
*O.lot*IR13	GAACGGCTCCAGGTAGATGT	GTGACCAACGAGAGGCTCTA	120
*O.lot*IR14	GGAGCTGGTGATGAACAAGAC	GCGGTGATGTAGTTGATGAGG	186
*O.lot*IR15	CGATGATGATGAAGCCGAAGAG	TACAAGGACGACGAGGAGAAG	157
*O.lot*IR16	ACACGACGGACGACTGGTA	GCATACACTACGGCATCAACTC	111
*O.lot*GADPH	CGCCTTTAACGAACATCGGAGT	TCGCTGAAGCAACTGGTCTGT	123
